# Cryptic diversity in smooth-shelled mussels on Southern Ocean islands: connectivity, hybridisation and a marine invasion

**DOI:** 10.1186/s12983-019-0332-y

**Published:** 2019-08-06

**Authors:** Małgorzata Zbawicka, Jonathan P. A. Gardner, Roman Wenne

**Affiliations:** 1 Institute of Oceanology, Polish Academy of Sciences, Powstańców Warszawy 55, 81-712 Sopot, Poland; 2 School of Biological Sciences, Victoria University of Wellington, P O Box 600, Wellington, 6140 New Zealand

**Keywords:** *Mytilus*, Southern Ocean, SNP genotyping, Introgression, Hybridisation, Falkland Islands, Kerguelen Islands, Tasmania, New Zealand

## Abstract

**Background:**

Large numbers of endemic species inhabit subantarctic continental coasts and islands that are characterised by highly variable environmental conditions. Southern hemisphere populations of taxa that are morphologically similar to northern counterparts have traditionally been considered to be extensions of such Northern hemisphere taxa, and may not exhibit differentiation amongst geographically isolated populations in the Southern Ocean. Smooth-shelled blue mussels of the genus *Mytilus* that exhibit an anti-tropical distribution are a model group to study phylogeography, speciation and hybridisation in the sea, and contribute to the theory and practice of marine biosecurity.

**Methods:**

We used a single nucleotide polymorphism (SNPs) panel that has the ability to accurately identify reference Northern and Southern hemisphere *Mytilus* taxa to test for evolutionary differentiation amongst native Southern Ocean island populations.

**Results:**

Native mussels from the Falkland Islands and the Kerguelen Islands exhibited greatest affinity to native *M. platensis* d’Orbigny 1846 from the Atlantic coast of South America. The major Southern Ocean current flow from west to east is likely to explain the spreading of *M. platensis* to remote offshore islands, as adults via the process of rafting or perhaps directly as larvae. SNPs variation revealed that mussels from Tasmania were native and clearly differentiated from all other blue mussel groups in the Southern and Northern hemispheres. The native mussels *M. planulatus* from Tasmania and from mainland New Zealand (NZ), and tentatively *M. aoteanus* from the two NZ Southern Ocean offshore island groups (the Auckland Islands and Campbell Island), formed a distinct *M. galloprovincialis*–like Southern hemisphere group with closest affinity to Northern hemisphere *M. galloprovincialis* from the Mediterranean Sea. In all cases, the SNPs revealed evidence of hybridisation between two or more distinct taxa. The invasive Northern hemisphere *M. galloprovincialis* was identified only in Tasmania, amongst native mussels of a distinct Australian *M. planulatus* lineage.

**Conclusion:**

Overall, our results reveal that Southern hemisphere island mussels have mixed genome ancestry and are native, not introduced by human activities. The preservation of distinct evolutionary lineages of Southern hemisphere species needs to be an ongoing focus of conservation efforts, given that population sizes on some of the remote offshore oceanic islands will be small and may be more easily adversely affected by invasion and subsequent hybridisation and introgression than larger populations elsewhere.

**Electronic supplementary material:**

The online version of this article (10.1186/s12983-019-0332-y) contains supplementary material, which is available to authorized users.

## Background

Subantarctic continental coasts and remote island intertidal zones are characterised by intermediate levels of biodiversity and large numbers of endemic species [1]. Morphologically similar Southern hemisphere populations have traditionally been considered to be extensions of Northern hemisphere populations and taxa, or undifferentiated populations in the Southern Ocean. Increasingly, over the last two or three centuries, anthropogenic activities have, however, resulted in the blurring of patterns of natural distributions, even in remote locations, often with profound ecological, economic and social costs [2–4]. Because of increased human activities, including maritime traffic, geographic ranges of endemic marine species may be extended, their populations mixed via hybridisation or endangered by invasions of alien species [5]. In addition, ocean rafting is increasingly recognised as an important natural method of range expansion of some marine taxa [6, 7] and there is evidence that increased storm activity resulting from global warming may help to break down the geographic isolation of regions such as Antarctica [8]. Whilst the natural distributions of some marine species may be relatively easy to identify in the absence of human-mediated accidental or deliberate movements, for many other species this may not be the case. Understanding the natural patterns of species distributions in the Southern Ocean have long been a challenge, given the scale of the endeavour, but this challenge is increasingly becoming more difficult due to recent mixing of species and the blurring of species’ natural distributions.

For some species, including smooth-shelled blue mussels of the genus *Mytilus*, the translocation of individuals may result in extensive erosion of species differences (genotypic and phenotypic) and the disruption of natural patterns of distribution, because such mussels are known to hybridise and backcross extensively in almost all cases where two or more species co-exist [9–11]. Invasive blue mussels, in particular the Northern hemisphere Mediterranean mussel, *Mytilus galloprovincialis*, occur in many regions of the world (refer to [4, 12, 13]) and in some regions, such as the Pacific coast of the United States, have out-competed and largely replaced the native species [14–16]. The mechanistic basis of this invasion success is presently unknown, but evidence points to the greater thermal tolerance of *M. galloprovincialis* over its congenerics [17].

Smooth-shelled blue mussels of the genus *Mytilus* are a model group that provide excellent opportunities to examine phylogeography, evolution, speciation and hybridisation in the sea and to test the theory and practice of marine biosecurity. Blue mussels are naturally widely occurring, with an anti-tropical distribution, in all areas except polar regions [12, 18]. They are remarkably tolerant of environmental variation [19], and are ecosystem engineers [20, 21]. In many parts of the world mussels are an important source of protein, both in terms of wild harvest and aquaculture production [22–24]. Over the last 50 years or so, a better global understanding of the phylogeography, taxonomy and systematics of smooth-shelled blue mussels of the genus *Mytilus* has been achieved with a range of genetic marker types, from allozymes (protein variation) [25–30], through mitochondrial DNA (mtDNA) sequencing and restriction fragment length polymorphisms (RFLPs) [31–42], to nuclear DNA markers [43–48], such as microsatellites [49–55] and, most recently, single nucleotide polymorphisms (SNPs) [56–61]. The development and application of each new generation of molecular marker type has provided new insight into the phylogeography and the biosecurity threat of blue mussels in a global perspective. The recent development of SNPs for mussels of the genus *Mytilus* [62] and their application to mussels from New Zealand [4], Chile [13] and Argentina [63] has revealed profound and consistent genetic differences amongst multiple Southern hemisphere evolutionary lineages. The SNPs data also confirm, in all of these regions, the existence of invasive Northern hemisphere *M. galloprovincialis* and/or the hybridisation and introgression of non-native genes into the native lineage.

Whilst the phylogeography and taxonomy of native Southern hemisphere blue mussels is becoming clearer following the application of the SNP markers, most of the work to date has focussed on collections from coastal sites on major land masses. One topic that has not been investigated in any depth is the question of the phylogeography of blue mussels on remote offshore islands in the southern Atlantic, Indian and Pacific oceans. These islands are very small land masses in a very large expanse of ocean that effectively encircles (below latitude 55°S) the Southern hemisphere, and which may provide some degree of connectivity for marine species with pelagic larval durations that are long enough to move from one stepping stone island to another [64–66] and/or may be capable of long distance movement via a process such as rafting on kelp [7, 67]. To date, the only SNPs investigation of New Zealand (NZ) Southern Ocean blue mussel island populations has highlighted the existence of unique island lineages and also introgression of non-native genes into island mussel populations [4]. Despite their remoteness, the NZ Southern Ocean islands do not have a refuge from invading mussels, and may, perhaps, act as a stepping stone for the introduction of invasive mussels, or at least for mussels with introgressed genes, to establish on Antarctic shores [68, 69]. These findings highlight the urgent need to describe and better understand the biogeography of blue mussels (and other marine taxa) found on the small and isolated islands of the Southern Ocean.

The aim of the present research was to determine the genetic differentiation, phylogeography and the taxonomic status of blue mussels from Southern Ocean islands and to investigate the extent, if any, of introduction of non-native blue mussels and their hybridisation and introgression with native species. We used SNPs that differentiate amongst mussel populations from the Southern hemisphere, including Argentina (*M. platensis*), Chile (*M. chilensis*) and New Zealand (Southern hemisphere *M. galloprovincialis*-like) and also amongst reference Northern hemisphere taxa (*M. edulis*, *M. galloprovincialis*, *M. trossulus*) [4, 13, 63]. We assayed SNP variation in mussels from the Falkland Islands (South Atlantic Ocean), Kerguelen Islands (South Indian Ocean) and from Tasmania (South Pacific Ocean). We also tested for the occurrence of hybridisation and introgression between native populations and invasive blue mussels. This works contributes to ongoing efforts to better understand the natural distributions, patterns of genetic connectivity across large ocean in scales and evolutionary affinities of blue mussels in the Southern hemisphere.

## Methods

### Sample collection and SNP genotyping

**Fig. 1:**
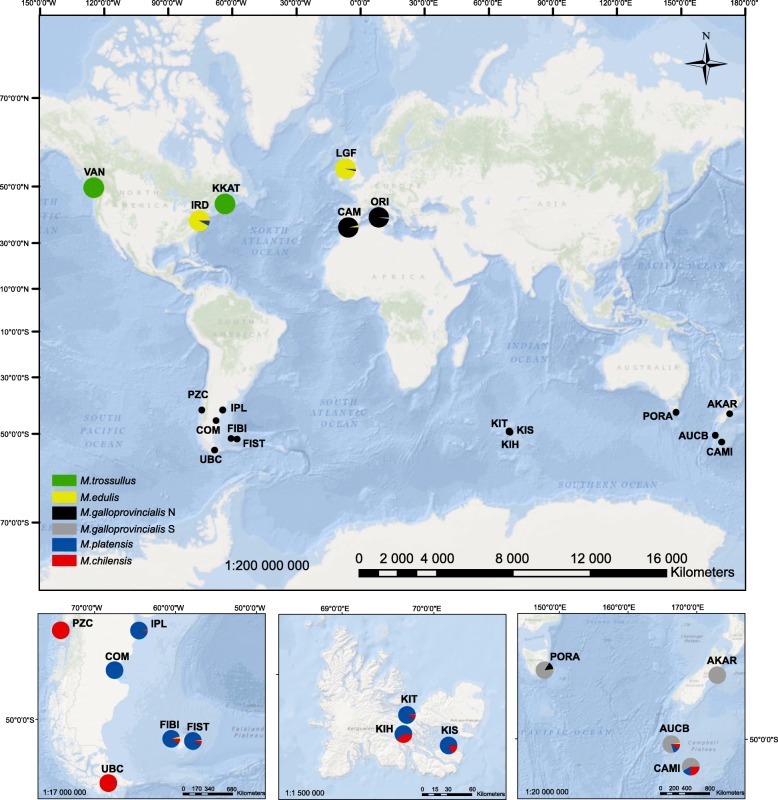
Location of the 19 samples of *Mytilus* from Falkland Islands, the Kerguelen Islands, Tasmania and reference samples from Northern and Southern hemisphere (ArcGIS). Sampling site names and coordinates are in Table 1. The pie charts visualise the proportions of the inferred clusters (K = 6) computed with Structure for 19 studied samples

*Mytilus* spp. samples that consisted of 77 individuals of mixed ages and sizes (5–50 mm shell length) were collected from six localities at the Falkland (Malvinas) Islands (2 sites), the Kerguelen Islands (3 sites: I3B, BO100av and IS in Gérard et al. [70]) and Tasmania (1 site) between 2002 and 2015 (Fig. 1, Table 1). Whole specimens or tissue samples were stored in 96% ethanol. DNA was isolated from the mantle tissue using a modified CTAB method [71]. Thirteen previously described reference samples including 354 specimens were included: *M. edulis* from the Atlantic coast of USA and Northern Ireland, UK; Northern hemisphere *M. galloprovincialis* from the Atlantic coast of Spain and the Mediterranean Sea; native Southern hemisphere *M. galloprovincialis*-like mussels from mainland New Zealand and from Southern Ocean offshore islands; *M. trossulus* from Atlantic Canada (Halifax, Nova Scotia) and Pacific Canada (Vancouver, British Columbia); *M. chilensis* from Chiloé, Chile and Ushuaia, Strait of Magellan, southern Argentina); and *M. platensis* from Comodoro Rivadavia on the Atlantic coast of Argentina [4, 13, 62, 63, 72]. To identify amongst populations and to identify instances of hybridisation, 79 SNPs that differentiate amongst species were used [4, 62, 63]. Samples were genotyped using the Sequenom MassARRAY iPLEX genotyping platform [73].

**Table 1:**
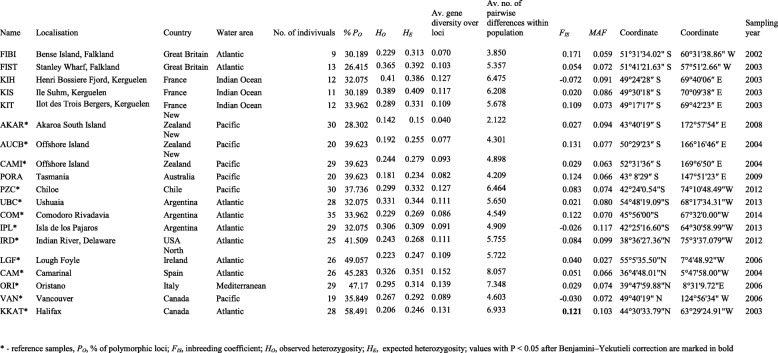
Localisation, number and genetic parameters of the 19 samples of *Mytilus* mussels

### Data analysis

#### Genetic diversity

Populations were analysed for allele frequencies, proportion of polymorphic SNPs (*P*_*O*_) genetic diversity, observed (*H*_*O*_) and expected (*H*_*E*_) heterozygosity, genetic differentiation (pairwise *F*_ST_), and inbreeding coefficient (*F*_IS_) using Arlequin v. 3.5.1.2 [74]. Departures from Hardy-Weinberg equilibrium (HWE) were tested by exact test, and significance was determined by Markov chain Monte Carlo simulations. The false discovery rate (FDR-BY) was used to correct significance (*P*) values after multiple testing [75, 76].

#### Population genetic differentiation and structure

*F*_ST_ distance measures in the Newick format, obtained in POPTREEW [77], were used to construct a neighbour-joining (NJ) tree illustrating the genetic relationships amongst populations. Correspondence analysis (CA; Benzécri [78]), implemented in GENETIX [79] was used to visualise genetic substructure amongst populations and individuals. Population structure was inferred by determining the number of clusters (groups) observed without prior knowledge of sampling location. Bayesian clustering using STRUCTURE v. 2.3.4 software with the model assuming admixture, ignoring population affiliation and allowing for the correlation of allele frequencies amongst clusters was used to infer groups [80, 81]. The most appropriate number of genetic clusters was determined by a diagram-based comparison of log-likelihoods for values of *K* ranging from one to the study number of populations plus one. At least five runs were used for each *K* value, following the method described by Evanno et al. [82]. The length of burn-in period was 50,000 and the number of MCMC cycles after burn-in was 100,000 iterations each. Genetic assignment was obtained by using two methods. Following the STRUCTURE analysis, a threshold value of q = 0.8 was used to assign individuals to clusters. Individuals with q-values from 0.2 to 0.8 were considered to be potentially admixed [83].

Genetic assignment of individuals to population of origin was carried out using frequency criteria on the basis of multilocus genotype data [84] and the Bayesian method of Rannala & Mountain [85] implemented in GeneClass2.0 [86]. Individuals were considered to be correctly assigned to their location of origin if the assignment probability to that group was higher than any other assignment probability to any other group.

#### Hybridisation and introgression

The likelihood of hybridisation and introgression was assessed using the software NewHybrids v1 [87]. NewHybrids was used to estimate the posterior probability that individuals from the Falkland Islands, the Kerguelen Islands, Tasmania and from the NZ Southern Ocean islands fell into one of the genotypic categories: *M. platensis*, *M. chilensis*, Northern hemisphere *M. galloprovincialis*, Southern hemisphere *M. galloprovincialis-*like, F1 hybrids, F2 hybrids and backcrosses.

## Results

### Genetic diversity

Eight samples from four regions (the Falkland Islands, the Kerguelen Islands, Tasmania, and the NZ Southern Ocean islands) encompassing the South Atlantic Ocean, the South Indian Ocean and the South Pacific Ocean were analysed for SNP variation, along with reference populations from both the Northern and Southern hemispheres. Of 79 SNPs assayed, 53 were used for analysis (Table 1, Additional file 2: Table S1 and Additional file 3: Table S2).

The proportion of polymorphic SNPs (*P*_*o*_) for the eight Southern hemisphere island populations ranged from 26.4 to 39.6%. Observed heterozygosity (*H*_*o*_) for 53 loci amongst most samples was lower than expected (*H*_*E*_). The samples from the Kerguelen Islands were characterised by high observed heterozygosity values and gene diversity values, compared to the samples from the NZ Southern Ocean islands (Table 1). Only nine of 1007 test results involving five different loci were not in Hardy-Weinberg equilibrium (HWE) after correction for multiple testing. *F*_ST_ values at individual SNP loci ranged from 0.028 to 1.000, and 26 SNPs had *F*_ST_ values significantly different from zero (Additional file 2: Table S1).

### Population genetic differentiation and structure

In total, 164 of 171 pairwise comparisons of *F*_ST_ values were significantly different from zero after FDR-BY correction. Four of the seven non-significant values involved samples from the Kerguelen Islands (Additional file 4: Table S3). Greatest differentiation was observed between the New Zealand *M. galloprovincialis*-like and the Canadian *M. trossulus* samples.

The Falkland Islands and Kerguelen Islands samples showed greatest similarity to reference *M. platensis* from Comodoro, Argentina (*F*_ST_ = 0.04–0.15). Despite this, the Falkland Islands and Kerguelen Islands mussels were significantly differentiated, based on *F*_ST_ values. The Tasmanian sample showed greatest similarity to the NZ mainland population of Akaroa (Southern hemisphere *M. galloprovincialis*-like) and then to the two Northern hemisphere *M. galloprovincialis* populations.

**Fig. 2:**
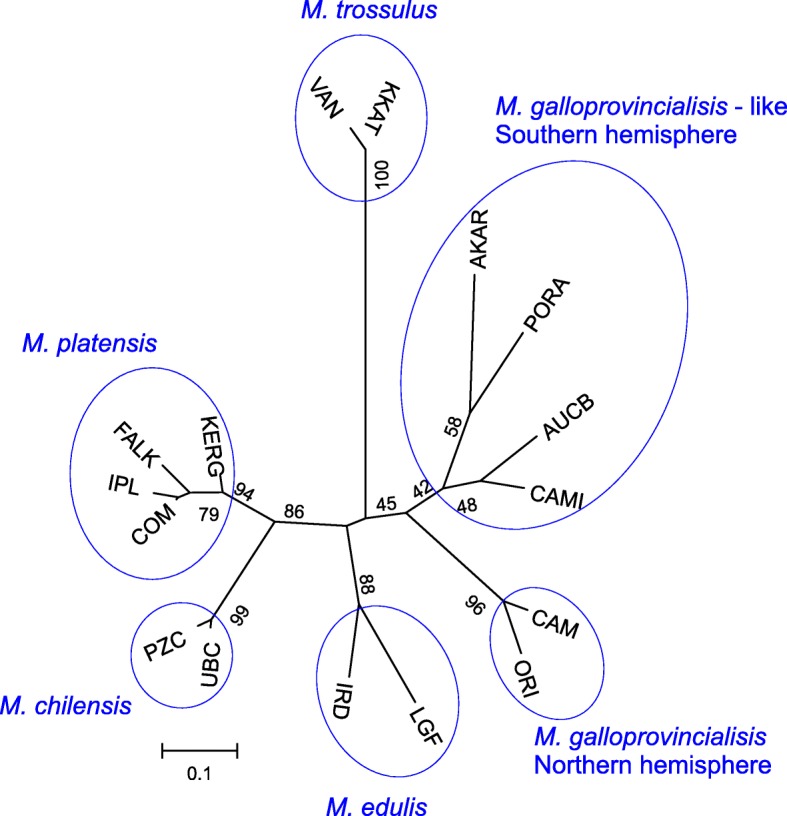
Neighbour-joining tree of Falkland Islands, the Kerguelen Islands, Tasmania and the reference *Mytilus* taxa. NJ tree shows genetic relationship between 16 *Mytilus* spp. samples based on the *F* distance measures obtained with POPTREEW and visualised with MEGA version 6

The NJ tree based on the *F*_ST_ distance matrix revealed six main clades: (1) *M. trossulus*, (2) Southern hemisphere *M. galloprovincialis*-like mussels, (3) Northern hemisphere *M. galloprovincialis*, (4) *M. edulis*, (5) *M. chilensis* from Chile and (6) *M. platensis* from Argentina, including mussels from the Falkland Islands and Kerguelen Islands (Fig. 2).

**Fig. 3:**
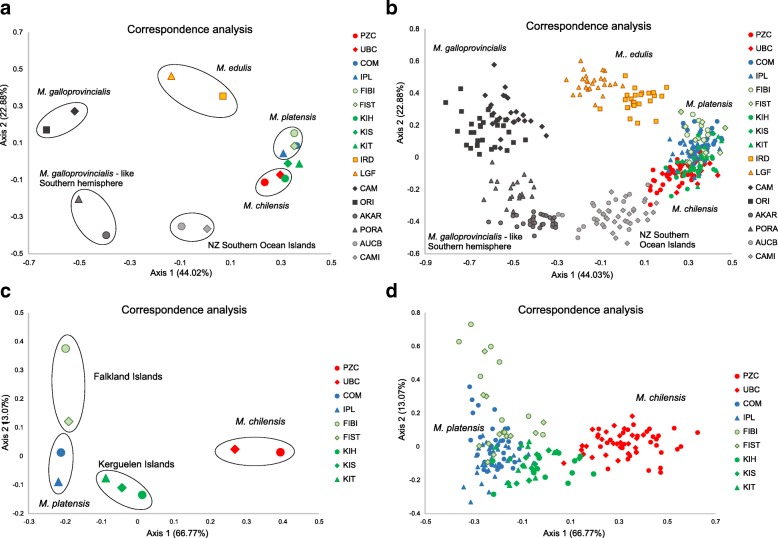
The first two axes of the correspondence analysis (CA) computed from the SNP data on sixteen samples from Falkland Islands, the Kerguelen Islands, Tasmania and reference populations of *M. edulis*, *M. galloprovincialis* and *M. chilensis* and *M. platensis* from America, Europe and New Zealand (**a** and **b**). CA of the Chile, Argentina, Falkland Islands and Kerguelen Islands mussels (**c** and **d**). Each dot is sample (**a** and **c**) or an individual (**b** and **d**)

Correspondence analyses (CA) carried out on 17 samples (i.e., excluding *M. trossulus*, for higher resolution of results) resolved five groups: the reference *M. edulis*, Southern hemisphere *M. galloprovincialis*-like, Northern hemisphere *M. galloprovincialis*, the NZ Southern Ocean islands, and *M. platensis* with *M. chilensis* including the Falkland Islands and Kerguelen Islands samples (Fig. 3a). The first two axes explained 66% of the total variation. Axis 1 (44% of variation) revealed a separation between *M. galloprovincialis*, *M. edulis* and *M. platensis* from *M. chilensis*. Axis 2 (22% of variation) revealed a separation between *M. galloprovincialis* populations from the Northern and Southern hemispheres and *M. platensis* from *M. chilensis*. CA carried out for individuals (Fig. 3b) revealed that mussels from the Kerguelen Islands exhibited overlap with the *M. platensis* and *M. chilensis* individuals, whereas mussels from the Falkland Islands exhibited far more overlap with *M. platensis* than with *M. chilensis*. Tasmanian mussels clustered together between Southern and Northern hemisphere *M. galloprovincialis*. NZ Southern Ocean island individuals clustered between Southern hemisphere *M. galloprovincialis*-like mussels from mainland NZ and *M. chilensis*. CA of the Chile, Argentina, Falkland Islands and Kerguelen Islands mussels (Fig. 3c) revealed clear separation of samples based on geography, with the Falkland Islands and Kerguelen Islands mussels showing greater similarity to *M. platensis* than to *M. chilensis*. The same CA based on individuals (Fig. 3d) revealed limited overlap of the Kerguelen Islands mussels with the *M. chilensis*, and no overlap of the Falkland Islands mussels with the *M. chilensis*. Considerable overlap was revealed for both the Falkland Islands and Kerguelen Islands mussels with *M. platensis*. Whilst axis 1 (66.8% of the total variation) explained the differentiation of *M. chilensis* from all other mussels, axis 2 (13.1%) contributed to explaining the separation amongst the Falkland Islands, Kerguelen Islands and *M. platensis* samples, although there was still some overlap amongst these individuals.

STRUCTURE analysis revealed the presence of six main clusters, although the largest increase in ΔK was obtained for *K* = 2, and then *K* = 3 and *K* = 6. For *K* = 2 only *M. trossulus* was separated from all other *Mytilus* taxa, whilst *K* = 3 clusters corresponded to *M. trossulus*, *M. edulis* together with *M. galloprovincialis*, and all other groups. Further subdivision was suggested due to the high value of ΔK for *K* = 6, where samples from Argentina (*M. platensis*), Chile and southern Argentina (*M. chilensis*), and New Zealand and Australia-Tasmania (Southern hemisphere *M. galloprovincialis*-like) were assigned to distinct clusters (Fig. 1, Additional file 1: Figure S1).

In STRUCTURE for *K* = 6 the reference individuals (*M. edulis*, *M. trossulus*, *M. galloprovincialis*, *M. chilensis* and *M. platensis*) were assigned to their original samples (taxa) with q values > 0.8. In contrast, individual assignments for Falkland Islands, Kerguelen Islands and Tasmania samples, which were influenced by introgression, were frequently intermediate with as much as 23, 25 and 20%, respectively, of samples showing q values between 0.2 and 0.8. Most individuals from the Falkland Islands and the Kerguelen Islands were assigned to *M. platensis*, whilst other individuals were considered potentially admixed (*M. chilensis* × *M. platensis*). Three individuals from the Kerguelen Islands population of Henri Bossiere Fjord clustered with *M. chilensis*. Individuals with genome admixture were assigned to two clusters, *M. chilensis* and *M. platensis*. In total, 75% of individuals from Tasmania were assigned to the Southern hemisphere *M. galloprovincialis*-like cluster, one individual (5%) was identified as non-native Northern hemisphere *M. galloprovincialis*, and the remaining individuals from Tasmania were identified as admixed, being assigned to Northern hemisphere *M. galloprovincialis* × Southern hemisphere *M. galloprovincialis*-like. The NZ Southern Ocean island mussels exhibited very high levels of admixture: 13 (27%) individuals were assigned to Southern hemisphere *M. galloprovincialis*-like with q values > 0.8, whereas all other individuals were assigned to two clusters, mainly to Southern hemisphere *M. galloprovincialis*-like × *M. chilensis* but also to Southern hemisphere *M. galloprovincialis*-like × *M. platensis*.

In total, 94% of individuals were correctly assigned to their location of origin using GeneClass2. Potentially admixed individuals from the Falkland Islands and the Kerguelen Islands were assigned to their original location or to the Argentinian sample (*M. platensis*) from Isla de los Pajaros (IPL). Likewise, most of the potentially admixed Tasmanian and NZ Southern Ocean islands individuals were assigned to their original location, or to mainland New Zealand (Additional file 5: Table S4 assignments).

### Hybridisation and introgression

In total, 14% of individuals from the Falkland Islands and 28% of individuals from the Kerguelen Islands were identified as F2 hybrids (*M. chilensis* × *M. platensis*). F1 hybrids and backcrosses were not detected in the Falkland Islands or Kerguelen Islands samples. In contrast, amongst mussels from the NZ Southern Ocean, 45% of Auckland Islands individuals and 90% of Campbell Island individuals were influenced by hybridisation and introgression. They were also identified as F2 hybrids (mainly Southern hemisphere *M. galloprovincialis*-like × *M. chilensis* or Southern hemisphere *M. galloprovincialis*-like × *M. platensis*). Hybrid and backcross individuals were not detected in the Tasmanian population.

## Discussion

### Phylogeography

Building on the recent descriptions of native blue mussels from Southern hemisphere locations, such as New Zealand [4], Chile [13] and Argentina [63], we show that mussels from the Southern Ocean islands are native and very different from the three recognised Northern hemisphere reference taxa of *M. edulis*, *M. galloprovincialis* and *M. trossulus*.

Native mussels from the Falkland Islands and the Kerguelen Islands showed greatest affinity to native mussels from the Atlantic coast of Argentina, that is, to *M. platensis* d’Orbigny, 1846 [63]. The separation of this group of South Atlantic Ocean and South Indian Ocean mussels is very well supported by the SNPs analyses that clearly differentiate it from all other blue mussel groups in the Northern and Southern hemispheres. Previously, Lamy [88] had recognised three species in this part of the world, based on shell morphological differences, including *M. chilensis* from Chile, *M. platensis* from Argentina and *M. desolationis* from the Kerguelen Islands. Subsequently, these mussels have been described as being *M. edulis*-like (that is, most similar to Northern hemisphere *M. edulis*) by a number of authors, based on a range of genetic markers and also on shell morphometric analyses, e.g. [26, 89, 90]. Here, a reasonably small panel of SNPs can correctly assign 91% of the Falkland Islands and also 91% of the Kerguelen Islands mussels (*M. platensis*-like) to their sampling group (native range), this being the highest value of correct assignment observed for all Southern hemisphere island blue mussel populations. In a broader perspective, when compared using the SNP markers to reference mussels from the Northern hemisphere, the *M. platensis* group shows greatest affinity to *M. edulis*, then to *M. galloprovincialis* and then to *M. trossulus*. At a broad level of interpretation, the SNPs-based results are consistent with earlier suggestions that *M. platensis* is “*M. edulis*-like”, but because of the high degree of species-specific definition across the SNPs panel, a quantifiable genetic difference exists amongst the four groups to allow us to differentiate *M. platensis* from *M. chilensis* and all Northern hemisphere species as a separate evolutionary lineage.

As assessed by SNP variation, the native mussels from Tasmania, from mainland New Zealand (NZ) and from the two groups of NZ Southern Ocean offshore islands (the Auckland Islands and the Campbell Islands) formed a group distinct from all other groups. This group has variously been called *M. planulatus* in Australia (Lamarck [91]), *M. aoteanus* or *M. edulis aoteanus* in New Zealand (Powell [92]), and Southern hemisphere *M. galloprovincialis* [93, 94]. McDonald et al. [26] had previously noted that mussels from Australia and NZ were similar in allozyme allele frequencies and shell morphology to Northern hemisphere *M. galloprovincialis*, and went on to note that such Southern hemisphere mussels are likely to be native, rather than introduced. The SNPs markers identify this group as being quite distinct from other Southern hemisphere mussels from both coasts of South America and from the other remote oceanic islands. Within this group, the mainland NZ population (Akaroa in the South Island) and the Australian sample from Port Arthur (Tasmania) called tentatively *M. planulatus* show greatest affinity and are differentiated from the NZ Southern Ocean remote island samples called tentatively *M. aoteanus*, with greater (e.g., the CA) or lesser (e.g., bootstrap values on the NJ tree) support. The Tasmanian, Campbell Island and Auckland Islands mussels can be assigned correctly to their sampling locations with 90, 83 and 80% accuracy. When compared to reference samples from the Northern hemisphere the mussels from this ‘Australasian’ group show greatest affinity to *M. galloprovincialis*, with the Tasmanian mussels showing greater affinity than the NZ mussels.

### Genetic connectivity and physical flow

The overall patterns of *Mytilus* Southern hemisphere island phylogeography as assessed using SNPs are remarkably consistent with the geography of sample locations, and with proximity to the major landmasses and their native mussels, as now reported using SNPs. This strongly suggests that natural long distance dispersal events have played a key role in the establishment of mussel populations on remote oceanic islands through the Southern Ocean.

Direction of ocean current in the Southern Ocean is from west to east, with a strong circular pattern of current at approx. 50–55° S that connects, to a greater or lesser extent, all land masses in the Southern hemisphere and many of the remote oceanic islands [95]. It is therefore not surprising that the native mussels of the Falkland Islands, or even of the Kerguelen Islands, show greatest affinity to the native mussels of Argentina (*M. platensis*), and that the different regions of the Southern hemisphere (Pacific coast of South America, Atlantic coast of South America, Australasia) are characterised by their own mussel lineages. As an aside, it is both interesting and surprising that South Africa alone as a major landmass in the Southern hemisphere was not colonised naturally by blue mussels (*Mytilus* sp. is a recent invader in South Africa - [96, 97]). This might be explained by the fact that southern Africa is in warm-temperate waters, north of the major Southern hemisphere west-to-east flow of cold-temperate and cold current that connects all other landmasses to the south [98].

Physical flow, and therefore connectivity in terms of pelagic larval dispersal (mussels typically have a 28 day pelagic larval duration which may be extended by cooler (oceanic) water temperatures – Bayne [99]), and/or rafting of juvenile or adult mussels on kelp [6, 100], will promote connectivity between regions. In contrast, major features such oceanic fronts may act as barriers to gene flow (connectivity) between regions, and may give rise to separate biogeographic faunas, both in shallow and deep waters (e.g. [98, 101–103] and references therein). Understanding the apparent conflicting effects of isolation caused by features such as oceanic fronts, which may give rise to different evolutionary lineages, and the promotion of gene flow via indirect mechanisms such as rafting, which may give rise to population-to-population connectivity or promote natural hybridisation between differentiated lineages, is a major challenge for an improved understanding of the marine biogeography of the Southern hemisphere. For example, oceanic fronts may act as barriers to gene flow over long (evolutionary) timescales, but may be semi-permeable (subject to jetting and incursions) at shorter timescales and in different places, e.g. [1, 104]. Indeed, there is increasing evidence in the Southern hemisphere of both cryptic speciation (different evolutionary lineages) of many groups of marine organisms, including crustaceans, echinoderms, molluscs, macroalgae and nematodes [1] and of long distance transport of kelps and kelp-associated organisms [8, 105]. Our results for Southern hemisphere blue mussels support this increasing body of biogeographic work and point to the importance of isolating features that may give rise to separate evolutionary lineages, and also the role that natural hybridisation may play in speciation on remote oceanic islands when connectivity is promoted, perhaps by a natural process such as rafting.

Because SNPs are co-dominant nuclear markers they are a powerful tool for the investigation of hybridisation and introgression that may occur, either naturally when two or more species/taxa share natural distributions [72, 106] or when one species (most often, Northern hemisphere *M. galloprovincialis*) has been introduced by human activity into the range of another, native species [4, 13, 16, 63]. Some SNP alleles that are characteristic of Northern hemisphere *M. trossulus* and *M. edulis*, but not of *M. galloprovincialis*, were observed in Southern hemisphere island populations, often at high frequency (e.g. BM101A, BM115B, BM12A, BM17B and BM61A - Additional file 3: Table S2). This situation has previously been reported for mussels from the NZ Southern Ocean islands [4]. SNP loci containing these alleles did not depart from Hardy-Weinberg Equilibrium, suggesting that these may be ancestral polymorphisms and that these loci may be evolutionarily conserved. However, a single specimen of Northern hemisphere *M. galloprovincialis* has been observed in Tasmania.

Most individuals identified as being potentially admixed individuals were indeed influenced by hybridisation and introgression. However, the extent of hybridisation and introgression varied from location to location. Although no F1 hybrids or backcross mussels were detected at either the Falkland Islands or the Kerguelen Islands, the Falkland Islands mussels showed a lower percentage of F2 mussels (*M. platensis* × *M. chilensis*) than did the Kerguelen Islands mussels (14% vs 28%), despite the latter being ~ 7800 km to the east, that is, further from the presumptive source of mainland South American populations. Putative hybrids of *M. chilensis* × Northern hemisphere *M. galloprovincialis* and also of *M. chilensis* × *M. trossulus* in several southern Chile mussel populations were reported with a single DNA marker (PCR-RFLP of Me 15–16) by Larraín et al. [107]. The restriction site of the RFLP-PCR Me15–16 assay [18, 108, 109] corresponds to SNP BM151A in this study. Allele T was found only in reference samples from Chile (*M. chilensis*) and New Zealand (*M. galloprovincialis*-like from Southern hemisphere), whereas allele G was found in the remaining locations (Northern hemisphere *M. edulis, M. galloprovincialis, M. trossulus* and Argentinian *M. platensis*). Thus, SNP BM151A cannot be treated as a diagnostic marker for Southern Hemisphere mussels.

Consistent with earlier work [4], but employing a new analytical approach, we identified high levels of hybridisation and introgression amongst mussels of the Auckland Islands (45%) and Campbell Island (90%), two remote NZ island groups separated by ~ 280 km in the Southern Ocean. All such mussels were F2 hybrids (no F1 hybrids or backcrosses were identified) of Southern hemisphere *M. galloprovincialis*-like mussels and either *M. chilensis* or *M. platensis*. In contrast, one invasive Northern hemisphere *M. galloprovincialis* individual was identified from Port Arthur (Tasmania, Australia) amongst native Southern hemisphere *M. galloprovincialis-*like mussels*.* Hybrids and backcrosses were not identified. Interpreting the Tasmanian results with published data need to be carried out with care, given that earlier studies have used different markers and samples from different locations, e.g. [18, 93, 94, 110–113].

## Conclusions

Native mussels from the Falkland Islands and the Kerguelen Islands showed greatest affinity to native mussels from the Atlantic coast of South America, that is, to *M. platensis*. The native mussels *M. planulatus*. From Tasmania and from mainland New Zealand (NZ), and tentative *M. aoteanus* from the two NZ Southern Ocean offshore islands (the Auckland Islands and Campbell Island) formed a *M. galloprovincialis*–like Southern hemisphere group. Invasive Northern hemisphere *M. galloprovincialis* was identified from Port Arthur (Tasmania, Australia). The application of SNPs markers to smooth-shelled blue mussel phylogeography and biosecurity illustrates the complexity of identifying invading individuals and/or introgressed non-native genes when population sizes may be small and rates of invasion or introgression are very low, and/or when the landscape of invasion is complex, with a limited number of sites (e.g., ports and marinas) experiencing invasion and most sites not. Such results highlight the need for ongoing monitoring of ports and marinas as points of entry for non-native species. It is hoped that a panel of SNPs can now be converted into diagnostic markers and used to rapidly and cheaply assay mussels and provide same-day results to managers who may need to understand the implications of bioinvasion and subsequent ecological and economic impacts on native mussels and the ecosystems in which they live. The preservation of distinct evolutionary lineages (or Southern hemisphere species as is increasingly becoming apparent) needs to be an ongoing focus of conservation efforts, given that population sizes on some of the remote offshore oceanic islands will be small and may be more easily adversely effected by invasion and subsequent hybridisation and introgression than larger populations elsewhere.

## Additional files

Additional file 1: **Figure S1.** Structure plots for the 19 studied samples (K = 6). Each individual is represented by a single vertical line broken into six coloured segments, with lengths proportional to each of the K inferred clusters. Abbreviation of the samples is provided in Table 1. Vertical black lines separate the populations. (PDF 1421 kb)

Additional file 2: **Table S1.** SNP properties, genome location, substitution type, *F*_ST_ *P*-value associated with test for outlier status, minor allele frequency. (PDF 302 kb)

Additional file 3: **Table S2.** Allele frequencies of 53 SNP loci for 19 *Mytilus* spp. samples. (PDF 190 kb)

Additional file 4: **Table S3.** *F*_ST_ distance matrix for 53 SNP, 19 samples of *Mytilus* mussels. (PDF 89 kb)

Additional file 5: **Table S4.** Result of population assignment algorithms GeneClass for 19 populations of mussels. (PDF 20 kb)

## Data Availability

Data are presented in Supplementary material.

## Abbreviations

mtDNA: Mitochondrial DNA
RFLPs: Restriction fragment length polymorphism
SNP: Single nucleotide polymorphism
