# Supplementary material for: Cryptic diversity in smooth-shelled mussels on Southern Ocean islands: connectivity, hybridisation and a marine invasion
Source: Front Zool. 2019 Aug 6;16:32. doi: 10.1186/s12983-019-0332-y (PMC6685288; doi:10.1186/s12983-019-0332-y)
Supplement: Supplementary file 1 — Figure S1. Structure plots for the 19 studied samples (K = 6). Each individual is represented by a single vertical line broken into six coloured segments, with lengths proportional to each of the K inferred clusters. Abbreviation of the samples is provided in Table 1. Vertical black lines separate the populations. (PDF 1421 kb) [file 12983_2019_332_MOESM1_ESM.pdf]

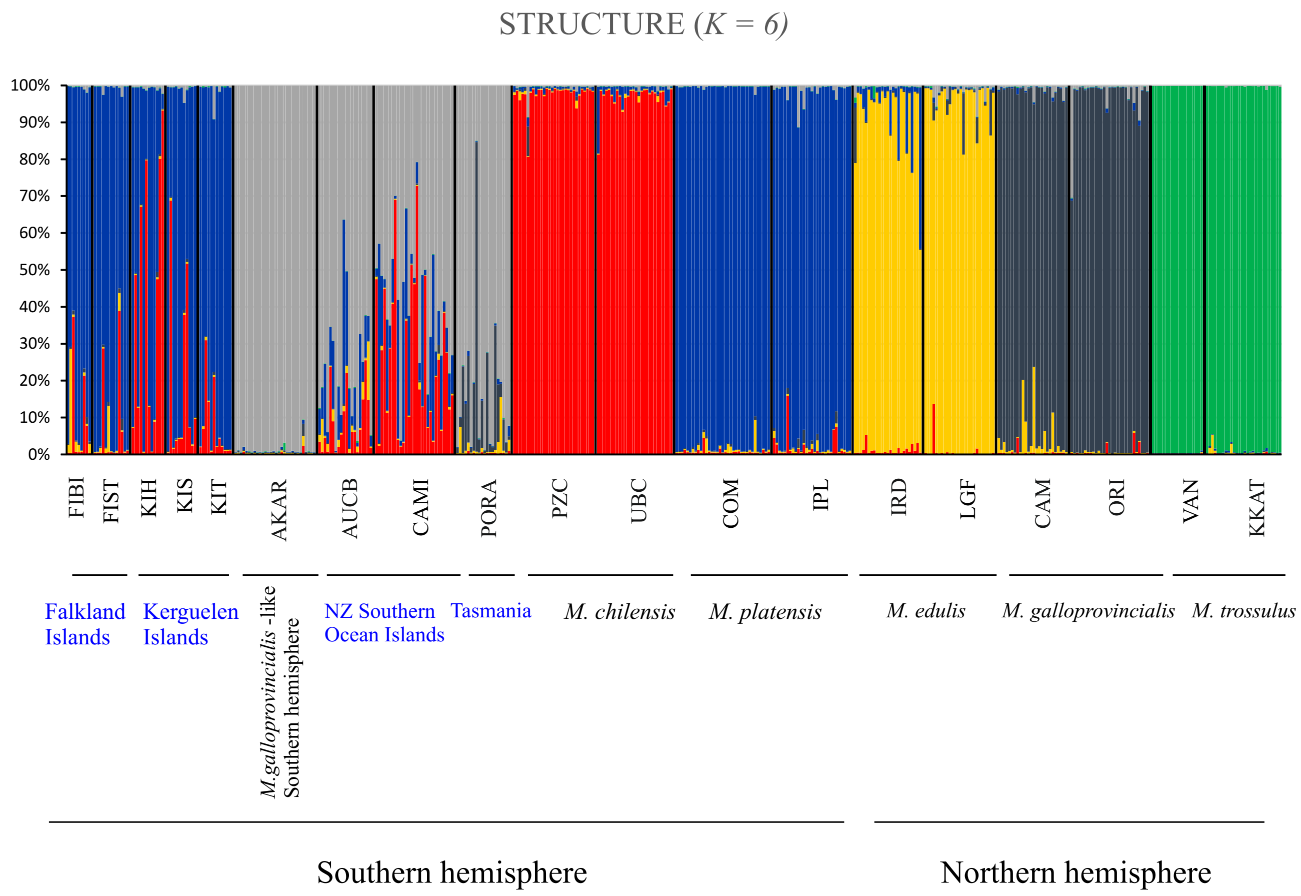

Figure S1. Structure plots for the 19 studied samples (K = 6). Each individual is represented by a single vertical line broken into six coloured segments, with lengths proportional to each of the K inferred clusters. Abbreviation of the samples is provided in Table 1. Vertical black lines separate the populations.
