# Supplementary material for: Cryptic diversity in smooth-shelled mussels on Southern Ocean islands: connectivity, hybridisation and a marine invasion
Source: Front Zool. 2019 Aug 6;16:32. doi: 10.1186/s12983-019-0332-y (PMC6685288; doi:10.1186/s12983-019-0332-y)
Supplement: Supplementary file 2 — Table S1. SNP properties, genome location, substitution type, FST P-value associated with test for outlier status, minor allele frequency. (PDF 302 kb) [file 12983_2019_332_MOESM2_ESM.pdf]

Table S1. SNP properties, genome location, substitution type, FST P-value associated with test for outlier status, minor allele frequency, GenBank annotation and references.

	Locus name	Location	Region	Substitution	Allele	Frequent allele	FST	HO	FIS	Nº of locations where the locus was polymorphic	MAF	GenBank annotation	Reference
1	BM101A	Ribosomal protein L7a	coding	synon	A/T	T	0.67	0.492	0.081	12	0.122	KT713378	Wenne et al. 2016
2	BM102A	Proteasome subunit beta type-6-like	coding	synon	C/T	T	**0.194** *	0.279	-0.079	15	0.164	KT713379	Wenne et al. 2016
3	BM103B	Proteasome subunit beta type-5-like	coding	nonsyn	A/G	A	0.735	0.158	-0.08	19	0.026	KT713380	Wenne et al. 2016
4	BM105A	UnKnown	NA	NA	A/G	A	0.413	0.128	0.157	4	0.053	KT713381	Wenne et al. 2016
5	BM106B	UnKnown	NA	NA	A/G	G	**0.862** *	0.566	**0.962**	19	0.060	KT713382	Wenne et al. 2016
6	BM10B	Ribosomal protein S20	coding	synon	A/C	A	0.875	0.184	0.028	19	0.012	KJ871040	Zbawicka et al.. 2014
7	BM113A	Protein BTG1	coding	synon	A/T	A	**1** *	1	0	0	0.000	KT713385	Wenne et al. 2016
8	BM115B	UnKnown	NA	NA	A/G	A	0.61	0.529	0.046	10	0.161	KT713384	Wenne et al. 2016
9	BM118A	UnKnown	NA	NA	A/G	G	**0.136**	0.062	-0.01	18	0.027	KT713388	Wenne et al. 2016
10	BM11A	Ribosomal protein L22	coding	synon	A/G	A	**0.977** *	0.206	-4E-04	2	0.002	KJ871041	Zbawicka et al.. 2014
11	BM12A	Ribosomal protein L23a	coding	synon	C/T	T	0.845	0.513	0.007	18	0.045	KJ871042	Zbawicka et al.. 2014
12	BM12C	Ribosomal protein L23a	coding	synon	C/T	C	0.678	0.528	0.024	18	0.103	KJ871042	Wenne et al. 2016
13	BM147A	UnKnown	NA	NA	C/T	C	**0.471** *	0.504	0.035	16	0.202	KT713383	Wenne et al. 2016
14	BM151A	adhesive foot protein gene	coding	synon	G/T	G	**0.864**	0.502	0.16	9	0.116	HQ257471.1	Gardner et al. 2016
15	BM16B	ribosomal protein L21	coding	synon	C/T	A	**0.044** *	0.32	-0.009	18	0.220	KJ871044	Wenne et al. 2016
16	BM17B	Ribosomal protein L7a	coding	synon	A/G	A	**0.582**	0.515	0.117	18	0.152	KJ871045	Zbawicka et al.. 2014
17	BM201B	*H4 histone* gene	noncoding	NA	A/C	C	0.777	0.162	**0.798**	19	0.021	AY267750.1	Zbawicka et al.. 2012
18	BM201C	*H4 histone* gene	coding	synon	G/T	T	0.604	0.288	0.057	9	0.065	AY267750.1	Zbawicka et al.. 2012
19	BM202A	*H3 histone* gene	noncoding	NA	A/C	A	**1** *	1	0	0	0.000	AY267749.1	Zbawicka et al.. 2012
20	BM202B	*H3 histone* gene	coding	synon	A/T	T	**1** *	1	0	0	0.000	AY267749.1	Zbawicka et al.. 2012
21	BM203B	*H2B histone* gene	noncoding	NA	C/T	C	0.59	0.286	0.054	9	0.067	AY267742.1	Zbawicka et al.. 2012
22	BM203D	*H2A histone* gene	noncoding	NA	A/T	A	**1** *	1	0	0	0.000	AY267757.1	Zbawicka et al.. 2012
23	BM204A	*p53*	coding	synon	C/T	T	**0.349**	0.18	0.058	7	0.070	DQ865151	Zbawicka et al.. 2012
24	BM21B	qm-like protein	coding	nonsyn	C/G	C	0.808	0.523	-0.071	16	0.059	KJ871047	Zbawicka et al.. 2014
25	BM21C	qm-like protein	coding	synon	A/C/T	A	0.677	0.152	0.002	16	NA	KJ871047	Zbawicka et al.. 2014
26	BM26B	UnKnown13	NA	NA	A/T	A	0.892	0.227	-0.128	2	0.011	KJ871050	Zbawicka et al.. 2014
27	BM2G	UnKnown05	coding	synon	G/T	G	**0.966** *	0.203	-0.036	19	0.003	KJ871032	Zbawicka et al.. 2014
28	BM30A	Ribosomal protein l17	coding	synon	A/G	A	0.864	0.182	-0.128	19	0.013	KJ871052	Zbawicka et al.. 2014
29	BM30C	Ribosomal protein l17	coding	synon	A/T	T	0.574	0.124	-0.014	19	0.042	KJ871052	Zbawicka et al.. 2014
30	BM32A	ubiquinol-cytochrome c reductase subunit 6	coding	synon	A/G	G	**0.169** *	0.497	**0.153**	18	0.329	KT713371	Wenne et al. 2016
31	BM33B	Cytochrome c oxidase subunit IV	coding	synon	A/T	A	0.416	0.099	-0.105	19	0.048	KJ871054	Zbawicka et al.. 2014
32	BM35C	Ribosomal protein L7	coding	synon	A/T	A	**0.369** *	0.508	0.131	18	0.228	KJ871055	Wenne et al. 2016
33	BM36F	ribosomal protein S3a	coding	synon	A/C	A	0.863	0.235	0.105	18	0.014	KT713373	Wenne et al. 2016
34	BM38B	ribosomal protein S8e	coding	synon	A/G	G	**0.394** *	0.48	0.048	18	0.203	KT713368	Wenne et al. 2016
35	BM44B	ubiquitin/ribosomal protein S27a	coding	synon	A/G	G	0.594	0.127	**0.416**	18	0.039	KJ871057	Zbawicka et al.. 2014
36	BM50B	CoA-binding protein	coding	synon	A/G	G	0.114	0.025	0.278	19	0.013	KJ871059	Zbawicka et al.. 2014
37	BM54A	ETC_C1_NDUFA4	coding	synon	A/G	A	**1** *	1	0	0	0.000	KJ871060	Zbawicka et al.. 2014
38	BM57A	NADH-ubiquinone_oxidoreductase	coding	nonsyn	C/T	C	0.56	0.337	0.095	7	0.123	KT713374	Wenne et al. 2016
39	BM57D	NADH-ubiquinone_oxidoreductase	coding	synon	A/C	C	0.525	0.133	-0.002	2	0.046	KT713374	Wenne et al. 2016
40	BM5B	Ribosomal protein S6e	coding	synon	A/G	A	0.079	0.039	-0.102	19	0.019	KJ871035	Wenne et al. 2016
41	BM5D	Ribosomal protein S6e	coding	synon	C/T	C	**0.263** *	0.175	-0.079	18.0	0.075	KJ871035	Zbawicka et al.. 2014
42	BM60A	UnKnown08	coding	synon	A/G		0.2	0.059	0.017	18	0.029	KJ871063	Zbawicka et al.. 2014
43	BM61A	Ribosomal_L1	coding	synon	C/T	T	**0.477** *	0.492	**0.281**	18	0.197	KT713375	Wenne et al. 2016
44	BM62A	Ribosomal L13e	coding	synon	A/G	G	0.828	0.241	0.219	6	0.025	KJ871064	Zbawicka et al.. 2014
45	BM64A	Ribosomal protein L35	coding	synon	C/T	C	**1** *	1	0	0	0.000	KJ871065	Zbawicka et al.. 2014
46	BM67C	Ribosomal protein S6e	coding	synon	A/T	T	**0.362** *	0.41	0.08	18	0.218	KJ871066	Wenne et al. 2016
47	BM6C	EFG_N	coding	synon	C/T	C	0.651	0.557	**0.179**	19	0.127	KJ871036	Zbawicka et al.. 2014
48	BM75C	UBA_UBA-TS-N_domain	coding	synon	C/G	C	0.028	0.014	-0.033	19	0.008	KT713370	Wenne et al. 2016
49	BM78B	UnKnown12	coding	synon	A/G	G	0.581	0.445	**0.184**	18	0.127	KJ871069	Zbawicka et al.. 2014
50	BM8E	Ribosomal protein L3	coding	synon	A/G	G	**0.989** *	0.208	0.003	1	0.001	KJ871038	Zbawicka et al.. 2014
51	BM92B	UnKnown06	coding	synon	A/T	A	**1** *	1	0	0	0.000	KJ871074	Zbawicka et al.. 2014
52	BM9B	Ribosomal protein S2	coding	synon	A/G	G	**0.203** *	0.471	**0.132**	18	0.277	KJ871039	Zbawicka et al.. 2014
53	BM9C	Ribosomal protein S2	coding	synon	A/C/T	A	**0.356** *	0.535	0.052	18	NA	KJ871039	Zbawicka et al.. 2014

Values with P < 0.05 are marked in bold; *, values with P < 0.05 after Benjamini–Yekutieli correction; $F_{IS,}$ inbreding coefficient; $H_{O,}$ observed heterozygosity; NA, not applicable
