# Supplementary material for: Cryptic diversity in smooth-shelled mussels on Southern Ocean islands: connectivity, hybridisation and a marine invasion
Source: Front Zool. 2019 Aug 6;16:32. doi: 10.1186/s12983-019-0332-y (PMC6685288; doi:10.1186/s12983-019-0332-y)
Supplement: Supplementary file 3 — Table S2. Allele frequencies of 53 SNP loci for 19 Mytilus spp. samples. (PDF 190 kb) [file 12983_2019_332_MOESM3_ESM.pdf]

TableS2. Allele frequencies of 53 SNP loci for 19 *Mytilus* spp.samples

Locus		Allele	FIBI	FIST	KIH	KIS	KIT	PZC	UBC	COM	IPL	IRD	LGF	CAM	ORI	AKAR	PORA	AUCB	CAMI	VAN	KKAT
BM101A	1	A	0.1667	0.1154	0.2083	0.2273	0.0455	0.4667	0.25	0	0.0714	0	0.04	1	1	1	0.975	0.4	0.6897	0	0
BM101A	2	T	0.8333	0.8846	0.7917	0.7727	0.9545	0.5333	0.75	1	0.9286	1	0.96	0	0	0	0.025	0.6	0.3103	1	1
BM102A	1	C	0.1429	0.1538	0.125	0.4545	0.125	0.3833	0.25	0.1	0.0345	0.54	0.3125	0.34	0.12	0	0.025	0	0	0.0909	0
BM102A	2	T	0.8571	0.8462	0.875	0.5455	0.875	0.6167	0.75	0.9	0.9655	0.46	0.6875	0.66	0.88	1	0.975	1	1	0.9091	1
BM103B	1	A	1	1	1	1	1	1	1	1	1	1	1	1	1	1	1	1	1	0.2368	0.2593
BM103B	2	G	0	0	0	0	0	0	0	0	0	0	0	0	0	0	0	0	0	0.7632	0.7407
BM105A	1	A	1	1	1	1	1	1	1	1	1	1	0.9808	0.4615	0.5714	1	0.9	1	1	1	1
BM105A	2	G	0	0	0	0	0	0	0	0	0	0	0.0192	0.5385	0.4286	0	0.1	0	0	0	0
BM106B	1	A	0.625	0.2308	0	0	0	0	0	0.1143	0	1	1	1	0.8571	0	0.75	0	0	1	0.9773
BM106B	2	G	0.375	0.7692	1	1	1	1	1	0.8857	1	0	0	0	0.1429	1	0.25	1	1	0	0.0227
BM10B	1	A	1	1	1	1	1	1	1	1	1	1	1	1	1	1	1	1	1	0.0278	0.1923
BM10B	2	C	0	0	0	0	0	0	0	0	0	0	0	0	0	0	0	0	0	0.9722	0.8077
BM113A	1	A	1	1	1	1	1	1	1	1	1	1	1	1	1	1	1	1	1	0	0
BM113A	2	T	0	0	0	0	0	0	0	0	0	0	0	0	0	0	0	0	0	1	1
BM115B	1	A	0	0.1538	0.3333	0.4545	0.1818	0.5172	0.375	0.2353	0.3036	1	1	1	1	1	1	0.4167	0.1176	0	0
BM115B	2	G	1	0.8462	0.6667	0.5455	0.8182	0.4828	0.625	0.7647	0.6964	0	0	0	0	0	0	0.5833	0.8824	1	1
BM118A	1	A	0	0	0	0	0	0	0	0	0	0	0.06	0.1	0.2586	0	0.05	0	0	0	0.0357
BM118A	2	G	1	1	1	1	1	1	1	1	1	1	0.94	0.9	0.7414	1	0.95	1	1	1	0.9643
BM11A	1	A	1	1	1	1	1	1	1	1	1	0.98	1	1	1	1	0.975	1	1	0	0
BM11A	2	G	0	0	0	0	0	0	0	0	0	0.02	0	0	0	0	0.025	0	0	1	1
BM12A	1	C	0.0625	0	0	0	0	0	0	0.0147	0	0.92	0.9808	0.9808	1	0.9833	1	0.8	0.3103	0	0.125
BM12A	2	T	0.9375	1	1	1	1	1	1	0.9853	1	0.08	0.0192	0.0192	0	0.0167	0	0.2	0.6897	1	0.875
BM12C	1	C	0.9	1	1	1	1	1	0.9643	0.9839	1	0.1087	0.0769	0.1923	0.7414	0	0.025	0.1154	0.7	0.6579	0.6154
BM12C	2	T	0.1	0	0	0	0	0	0.0357	0.0161	0	0.8913	0.9231	0.8077	0.2586	1	0.975	0.8846	0.3	0.3421	0.3846
BM147A	1	C	0.8571	0.5	0.6364	0.8182	0.5833	0.4667	0.6786	0.9	0.8621	1	0.9	0.9231	0.7241	0.2	0.3333	0.0263	0.1897	0	0
BM147A	2	T	0.1429	0.5	0.3636	0.1818	0.4167	0.5333	0.3214	0.1	0.1379	0	0.1	0.0769	0.2759	0.8	0.6667	0.9737	0.8103	1	1
BM151A	1	G	1	1	0.4167	0.7273	0.8333	0	0	0.9857	0.8966	1	0.98	1	1	0	0.1667	0.0263	0.0179	1	1
BM151A	2	T	0	0	0.5833	0.2727	0.1667	1	1	0.0143	0.1034	0	0.02	0	0	1	0.8333	0.9737	0.9821	0	0
BM16B	1	A	0.7222	0.6538	0.5833	0.5455	0.6667	0.7333	0.7679	0.8143	0.8621	0.84	0.76	0.7885	0.7931	0.95	0.85	0.85	0.7321	1	0.9107
BM16B	2	G	0.2778	0.3462	0.4167	0.4545	0.3333	0.2667	0.2321	0.1857	0.1379	0.16	0.24	0.2115	0.2069	0.05	0.15	0.15	0.2679	0	0.0893
BM17B	1	A	0.8333	0.8846	0.7917	0.7727	0.9583	0.5333	0.75	1	0.931	0.84	0.7308	0	0	0	0.025	0.6	0.3103	0.0714	0.1
BM17B	2	G	0.1667	0.1154	0.2083	0.2273	0.0417	0.4667	0.25	0	0.069	0.16	0.2692	1	1	1	0.975	0.4	0.6897	0.9286	0.9
BM201B	1	A	0	0	0	0	0	0	0	0	0	0	0	0	0	0	0	0	0	0.8889	0.7115
BM201B	2	C	1	1	1	1	1	1	1	1	1	1	1	1	1	1	1	1	1	0.1111	0.2885
BM201C	1	G	0	0	0	0	0	0.0167	0	0	0	0.02	0.0577	0.62	0.8571	0.3276	0.8158	0.0789	0.0345	0	0
BM201C	2	T	1	1	1	1	1	0.9833	1	1	1	0.98	0.9423	0.38	0.1429	0.6724	0.1842	0.9211	0.9655	1	1
BM202A	1	A	1	1	1	1	1	1	1	1	1	1	1	1	1	1	1	1	1	0	0
BM202A	2	C	0	0	0	0	0	0	0	0	0	0	0	0	0	0	0	0	0	1	1
BM202B	1	A	0	0	0	0	0	0	0	0	0	0	0	0	0	0	0	0	0	1	1
BM202B	2	T	1	1	1	1	1	1	1	1	1	1	1	1	1	1	1	1	1	0	0
BM203B	1	C	1	1	1	1	1	0.9833	1	1	1	0.98	0.9423	0.3696	0.1852	0.6607	0.2	0.9444	0.9655	1	1
BM203B	2	T	0	0	0	0	0	0.0167	0	0	0	0.02	0.0577	0.6304	0.8148	0.3393	0.8	0.0556	0.0345	0	0
BM203D	1	A	1	1	1	1	1	1	1	1	1	1	1	1	1	1	1	1	1	0	0
BM203D	2	T	0	0	0	0	0	0	0	0	0	0	0	0	0	0	0	0	0	1	1
BM204A	1	C	0	0	0	0	0	0	0	0	0	0.1	0.0385	0.36	0.6429	0.15	0.25	0.075	0	0	0
BM204A	2	T	1	1	1	1	1	1	1	1	1	0.9	0.9615	0.64	0.3571	0.85	0.75	0.925	1	1	1
BM21B	1	C	1	1	1	1	0.95	0.4167	0.6071	0.9857	0.9464	0.94	0	0	0	0	0	0.025	0.0862	1	0.9821
BM21B	2	G	0	0	0	0	0.05	0.5833	0.3929	0.0143	0.0536	0.06	1	1	1	1	1	0.975	0.9138	0	0.0179
BM21C	1	A	1	1	1	1	1	1	1	1	1	0.94	0.04	0.9231	0.8966	0.9833	0.925	1	0.9828	1	0.9821
BM21C	2	C	0	0	0	0	0	0	0	0	0	0.06	0.94	0.0192	0	0	0.05	0	0	0	0.0179
BM21C	3	T	0	0	0	0	0	0	0	0	0	0	0.02	0.0577	0.1034	0.0167	0.025	0	0.0172	0	0
BM26B	1	A	1	1	1	1	1	1	1	1	1	1	1	0.9423	0.8448	1	1	1	1	0	0
BM26B	2	T	0	0	0	0	0	0	0	0	0	0	0	0.0577	0.1552	0	0	0	0	1	1
BM2G	1	G	1	1	1	1	1	1	1	1	1	1	1	1	1	1	1	1	1	0	0.0556
BM2G	2	T	0	0	0	0	0	0	0	0	0	0	0	0	0	0	0	0	0	1	0.9444
BM30A	1	A	1	1	1	1	1	1	1	1	1	1	1	1	1	1	1	1	1	0.1053	0.1429
BM30A	2	G	0	0	0	0	0	0	0	0	0	0	0	0	0	0	0	0	0	0.8947	0.8571
BM30C	1	A	0	0	0	0	0	0	0	0	0	0	0	0	0	0	0	0	0	0.6842	0.5179
BM30C	2	T	1	1	1	1	1	1	1	1	1	1	1	1	1	1	1	1	1	0.3158	0.4821
BM32A	1	A	0.125	0.4231	0.5417	0.5556	0.4545	0.3667	0.3519	0.4853	0.4138	0.32	0.5577	0.4	0.5385	0.9107	0.4211	0.8421	0.8036	0.1111	0.1296
BM32A	2	G	0.875	0.5769	0.4583	0.4444	0.5455	0.6333	0.6481	0.5147	0.5862	0.68	0.4423	0.6	0.4615	0.0893	0.5789	0.1579	0.1964	0.8889	0.8704
BM33B	1	A	1	1	1	1	1	1	1	1	1	1	1	1	1	0.9821	1	1	1	0.5588	0.5417
BM33B	2	T	0	0	0	0	0	0	0	0	0	0	0	0	0	0.0179	0	0	0	0.4412	0.4583
BM35C	1	A	0.8571	0.9231	0.7083	0.9091	0.7083	0.3	0.3889	0.8824	0.9138	0.52	0.5833	0.38	0.2759	0.0667	0.4737	0.1579	0.2143	1	0.9286
BM35C	2	T	0.1429	0.0769	0.2917	0.0909	0.2917	0.7	0.6111	0.1176	0.0862	0.48	0.4167	0.62	0.7241	0.9333	0.5263	0.8421	0.7857	0	0.0714
BM36F	1	A	1	1	1	1	1	1	1	1	1	0.94	0.9348	0.9615	0.9107	1	1	1	1	0	0.0179
BM36F	2	C	0	0	0	0	0	0	0	0	0	0.06	0.0652	0.0385	0.0893	0	0	0	0	1	0.9821
BM38B	1	A	0.2143	0	0.4167	0.35	0.2273	0.1552	0.1071	0.2424	0.1724	0.04	0.0769	0.1346	0.2759	0.9333	0.475	0.8	0.6379	0.9474	0.7143
BM38B	2	G	0.7857	1	0.5833	0.65	0.7727	0.8448	0.8929	0.7576	0.8276	0.96	0.9231	0.8654	0.7241	0.0667	0.525	0.2	0.3621	0.0526	0.2857
BM44B	1	A	0	0	0	0	0	0	0	0	0	0	0	0	0	0	0	0	0	0.7188	0.537
BM44B	2	G	1	1	1	1	1	1	1	1	1	1	1	1	1	1	1	1	1	0.2812	0.463
BM50B	1	A	0	0	0	0	0	0	0	0	0	0	0	0	0	0	0	0	0	0.1842	0.0714
BM50B	2	G	1	1	1	1	1	1	1	1	1	1	1	1	1	1	1	1	1	0.8158	0.9286
BM54A	1	A	1	1	1	1	1	1	1	1	1	1	1	1	1	1	1	1	1	0	0
BM54A	2	G	0	0	0	0	0	0	0	0	0	0	0	0	0	0	0	0	0	1	1
BM57A	1	C	1	1	0.5417	0.6818	0.75	0.1607	0.375	1	1	1	1	0.4231	0.3462	1	1	1	1	1	1
BM57A	2	T	0	0	0.4583	0.3182	0.25	0.8393	0.625	0	0	0	0	0.5769	0.6538	0	0	0	0	0	0
BM57D	1	A	0	0	0	0	0	0	0	0	0	0	0	0.4808	0.6071	0	0	0	0	0	0
BM57D	2	C	1	1	1	1	1	1	1	1	1	1	1	0.5192	0.3929	1	1	1	1	1	1
BM5B	1	A	1	1	1	1	1	1	1	1	1	1	1	1	0.8966	1	0.975	1	1	0.8421	0.9286
BM5B	2	G	0	0	0	0	0	0	0	0	0	0	0	0	0.1034	0	0.025	0	0	0.1579	0.0714
BM5D	1	C	1	1	1	1	1	0.9667	1	1	1	0.8	0.4038	0.7885	0.8621	1	0.95	1	1	0.8158	0.8036
BM5D	2	T	0	0	0	0	0	0.0333	0	0	0	0.2	0.5962	0.2115	0.1379	0	0.05	0	0	0.1842	0.1964
BM60A	1	A	0	0	0	0	0	0	0	0	0	0	0	0	0	0.0185	0	0.0263	0	0.2632	0.25
BM60A	2	G	1	1	1	1	1	1	1	1	1	1	1	1	1	0.9815	1	0.9737	1	0.7368	0.75
BM61A	1	C	0.375	0.4167	0.3333	0.2727	0.2083	0.8913	0.9259	0.4375	0.2885	0.44	0.6522	0.84	0.9259	0	0	0	0.0345	0	0.1786

BM61A	2	T	0.625	0.5833	0.6667	0.7273	0.7917	0.1087	0.0741	0.5625	0.7115	0.56	0.3478	0.16	0.0741	1	1	1	0.9655	1	0.8214
BM62A	1	A	0	0	0.0417	0.0909	0.0833	0.1833	0.0357	0	0	0.04	0	0	0	0	0	0	0	1	1
BM62A	2	G	1	1	0.9583	0.9091	0.9167	0.8167	0.9643	1	1	0.96	1	1	1	1	1	1	1	0	0
BM64A	1	C	1	1	1	1	1	1	1	1	1	1	1	1	1	1	1	1	1	0	0
BM64A	2	T	0	0	0	0	0	0	0	0	0	0	0	0	0	0	0	0	0	1	1
BM67C	1	A	0.625	0.5385	0.375	0.2273	0.4167	0.1333	0.0536	0.5441	0.4655	0.7292	0.72	0.5769	0.0345	0	0	0.1	0.0172	0	0.0536
BM67C	2	T	0.375	0.4615	0.625	0.7727	0.5833	0.8667	0.9464	0.4559	0.5345	0.2708	0.28	0.4231	0.9655	1	1	0.9	0.9828	1	0.9464
BM6C	1	C	1	1	0.9583	1	1	0.9833	1	0.8857	0.4643	0.36	0.0962	0	0	0	0	0.2222	0.6562	0.6667	0.4286
BM6C	2	T	0	0	0.0417	0	0	0.0167	0	0.1143	0.5357	0.64	0.9038	1	1	1	1	0.7778	0.3438	0.3333	0.5714
BM75C	1	C	1	1	1	1	0.95	1	1	1	1	1	1	1	1	1	1	1	1	0.9667	0.9375
BM75C	2	G	0	0	0	0	0.05	0	0	0	0	0	0	0	0	0	0	0	0	0.0333	0.0625
BM78B	1	A	0.2143	0.25	0	0	0	0	0	0.069	0.1739	0	0.1538	0.56	0.7931	0.9833	0.9474	0.6333	0.4231	0	0.037
BM78B	2	G	0.7857	0.75	1	1	1	1	1	0.931	0.8261	1	0.8462	0.44	0.2069	0.0167	0.0526	0.3667	0.5769	1	0.963
BM8E	1	A	0	0	0	0	0	0	0	0	0	0	0.0192	0	0	0	0	0	0	1	1
BM8E	2	G	1	1	1	1	1	1	1	1	1	1	0.9808	1	1	1	1	1	1	0	0
BM92B	1	A	1	1	1	1	1	1	1	1	1	1	1	1	1	1	1	1	1	0	0
BM92B	2	T	0	0	0	0	0	0	0	0	0	0	0	0	0	0	0	0	0	1	1
BM9B	1	A	0.8889	0.5769	0.1667	0.3	0.375	0.65	0.5893	0.4714	0.4107	0.6304	0.5476	0.2885	0.1429	0.0167	0	0.15	0.1897	0.3333	0.3
BM9B	2	G	0.1111	0.4231	0.8333	0.7	0.625	0.35	0.4107	0.5286	0.5893	0.3696	0.4524	0.7115	0.8571	0.9833	1	0.85	0.8103	0.6667	0.7
BM9C	1	A	0.1111	0.4231	0.8333	0.6818	0.5833	0.3	0.2857	0.5147	0.5357	0.24	0.3	0.7115	0.8704	0.9833	1	0.825	0.8103	0	0.0357
BM9C	2	C	0	0.0769	0	0.0909	0.0833	0	0.0179	0.0441	0.0536	0.14	0.08	0	0	0	0	0	0	0	0
BM9C	3	T	0.8889	0.5	0.1667	0.2273	0.3333	0.7	0.6964	0.4412	0.4107	0.62	0.62	0.2885	0.1296	0.0167	0	0.175	0.1897	1	0.9643
