# Supplementary material for: Cryptic diversity in smooth-shelled mussels on Southern Ocean islands: connectivity, hybridisation and a marine invasion
Source: Front Zool. 2019 Aug 6;16:32. doi: 10.1186/s12983-019-0332-y (PMC6685288; doi:10.1186/s12983-019-0332-y)
Supplement: Supplementary file 4 — Table S3. FST distance matrix for 53 SNP, 19 samples of Mytilus mussels. (PDF 89 kb) [file 12983_2019_332_MOESM4_ESM.pdf]

Table S3. FST distance matrix for 53 SNP, 19 samples of *Mytilus* mussels

	FIBI	FIST	KIH	KIS	KIT	AKAR	AUCB	CAMI	PORA	PZC	UBC	COM	IPL	IRD	LGF	CAM	ORI	VAN
FIST	0.072																	
KIH	**0.261**	**0.153**																
KIS	**0.227**	**0.113**	0.016															
KIT	**0.171**	0.042	0.028	0.028														
AKAR	**0.810**	**0.774**	**0.714**	**0.739**	**0.755**													
AUCB	**0.594**	**0.546**	**0.442**	**0.495**	**0.489**	**0.319**												
CAMI	**0.518**	**0.469**	**0.334**	**0.392**	**0.395**	**0.406**	**0.117**											
PORA	**0.698**	**0.671**	**0.622**	**0.637**	**0.661**	**0.286**	**0.391**	**0.437**										
PZC	**0.349**	**0.338**	**0.217**	**0.252**	**0.273**	**0.653**	**0.446**	**0.338**	**0.595**									
UBC	**0.319**	**0.301**	**0.187**	**0.227**	**0.236**	**0.705**	**0.492**	**0.392**	**0.641**	**0.030**								
COM	**0.132**	**0.043**	**0.155**	**0.110**	**0.049**	**0.755**	**0.561**	**0.495**	**0.690**	**0.388**	**0.326**							
IPL	**0.180**	**0.085**	**0.158**	**0.126**	**0.080**	**0.727**	**0.519**	**0.448**	**0.650**	**0.376**	**0.327**	0.041						
IRD	**0.308**	**0.372**	**0.446**	**0.414**	**0.418**	**0.715**	**0.553**	**0.570**	**0.599**	**0.495**	**0.488**	**0.399**	**0.400**					
LGF	**0.501**	**0.515**	**0.552**	**0.538**	**0.538**	**0.687**	**0.538**	**0.569**	**0.564**	**0.548**	**0.562**	**0.545**	**0.527**	**0.257**				
CAM	**0.529**	**0.547**	**0.536**	**0.529**	**0.555**	**0.547**	**0.512**	**0.526**	**0.327**	**0.518**	**0.557**	**0.596**	**0.564**	**0.423**	**0.382**			
ORI	**0.596**	**0.594**	**0.566**	**0.563**	**0.589**	**0.547**	**0.528**	**0.535**	**0.330**	**0.541**	**0.581**	**0.633**	**0.599**	**0.540**	**0.504**	**0.107**		
VAN	**0.807**	**0.799**	**0.789**	**0.791**	**0.793**	**0.895**	**0.830**	**0.813**	**0.856**	**0.788**	**0.805**	**0.814**	**0.806**	**0.806**	**0.820**	**0.802**	**0.813**	
KKAT	**0.734**	**0.743**	**0.742**	**0.742**	**0.741**	**0.850**	**0.782**	**0.774**	**0.807**	**0.757**	**0.768**	**0.775**	**0.765**	**0.760**	**0.776**	**0.766**	**0.779**	0.024

Values with P<0.05 after Benjamini–Yekutieli (FDR-BY) correction is marked in bold. See Table 1 for site name definition.
