# Supplementary material for: Cryptic diversity in smooth-shelled mussels on Southern Ocean islands: connectivity, hybridisation and a marine invasion
Source: Front Zool. 2019 Aug 6;16:32. doi: 10.1186/s12983-019-0332-y (PMC6685288; doi:10.1186/s12983-019-0332-y)
Supplement: Supplementary file 5 — Table S4. Result of population assignment algorithms GeneClass for 19 populations of mussels. (PDF 20 kb) [file 12983_2019_332_MOESM5_ESM.pdf]

Table S4. Result of population assignment algorithms GeneClass for 19 populations of mussels.

		GeneClass		Assigned to species			
No. of indivivuals	Name	*M. platensis*	*M. chilensis*	*M. edulis*	*M. galloprovincialis - like* Southern Hemisphere	*M. galloprovincialis* Northern Hemisphere	*M. trossulus*
9	FIBI	9					
13	FIST	13					
12	KIH	6	6				
11	KIS	10	1				
12	KIT	12					
30	AKAR				30		
20	AUCB		6		14		
29	CAMI	3	15		11		
20	PORA				19	1	
30	PZC		30				
28	UBC		28				
35	COM	35					
29	IPL	29					
25	IRD			25			
26	LGF			26			
26	CAM					26	
29	ORI					29	
19	VAN						19
28	KKAT						28

GeneClass Assigned of individuals to origin region

	Falkland		Kerguelen			New Zealand	NZ Southern Ocean islands		Tasmania	Chile	Argentina	Argentina		USA	North Ireland	Spain	Italy	Canada	
	FIBI	FIST	KIH	KIS	KIT	AKAR	AUCB	CAMI	PORA	PZC	UBC	COM	IPL	IRD	LGF	CAM	ORI	VAN	KKAT
FIBI	7	2																	
FIST	1	10										1	1						
KIH			10										2						
KIS			1	10															
KIT			1		10								1						
AKAR						30													
AUCB						1	16	3											
CAMI						1	4	24											
PORA						2			18										
PZC										21	9								
UBC			1							4	23								
COM	1				1							32	1						
IPL			1									3	25						
IRD														25					
LGF															26				
CAM																25	1		
ORI																1	28		
VAN																		19	
KKAT																		2	26
